# Fish farming as an innovative strategy for promoting food security in drought risk regions of Zimbabwe

**DOI:** 10.4102/jamba.v9i1.491

**Published:** 2017-11-28

**Authors:** Elvin Shava, Constance Gunhidzirai

**Affiliations:** 1 Department of Public Administration, University of Fort Hare, South Africa; 2 Department of Social Work and Social Development, University of Fort Hare, South Africa

## Abstract

This article examines the implementation of fish farming as an innovative and economic strategy for promoting food security and dietary diversities among vulnerable households in drought risk areas of Zimbabwe. The declining climatic conditions and lack of economic opportunities in Mwenezi district of Zimbabwe attracted the attention of three non-governmental organisations (NGOs) to implement fish farming as an innovative mechanism to stimulate food security and generate employment in the district. The article used a qualitative research approach that includes semi-structured interviews and secondary data. The purposive sampling technique was adopted to interview participants in Mwenezi district who were involved in fish farming to assess and explore the experiences and benefits they derive from such development projects. Results for the article revealed that fish farming was well embraced by local communities as it led to improvements in food security, household income and employment regeneration. The local government including traditional leadership (Chiefs and Headmen’s) supported the NGO activities as they benefited local communities. The article concludes that although fish farming was instrumental in regenerating employment, some participants still fail to participate because of laziness and desire to maintain dependency syndrome. The article recommends the NGOs to launch awareness campaigns in rural communities and increase networking with the donor community which is fundamental in attracting sustainable funding. The government can also promote fish farming in vulnerable rural communities by providing funding and capacity building programmes.

## Introduction

This article introduces fish farming, also known as aquaculture as an economic and innovative technique for enhancing food security in drought prone regions of Zimbabwe. Studies (Food Agricultural Organisation 2011; Jamu & Brummett 2004) assert that aquaculture entered the global prominence at a time when governments were striving to ensure food security among communities. Jamu and Brummett (2004) explain that aquaculture is regarded as the practise of farming aquatic plants and animals such as fish, molluscs, crustaceans and aquatic plants in a modified environment. Farming entails the forms of intervention in the rearing process to enhance production including regular stocking, feeding and protection from predators. Mwaijande and Lugendo (2015) describe fish farming as an approach for economic transformation and poverty alleviation. It focuses on mitigating the significant barriers faced by fish farmers, traders, processors and other related actors in the value chain. This inevitably includes various activities such as ensuring success to a full range of necessary resources, inputs and technology. In Zimbabwe, fish farming is mainly spearheaded by non-governmental organisations (NGOs), as an economic mechanism for generating employment and increasing food security in vulnerable communities. Fish farming is fast gaining momentum among other rural livelihoods such as agriculture because of its untapped potential to generate employment and improve food security as it provides highly nutritious animal protein and important micronutrients among vulnerable households (FAO 2012). Climate change in Zimbabwe has caused food insecurity by reducing crop yields and increasing production variability (Devendra 2012). Swaminathan and Kesavan (2012) contend that the ever-increasing extreme weather conditions have negative effects on water, food and livelihood security in sub-Saharan countries where hunger and poverty are widespread. Studies (Codjoe & Owe 2011; Ringler et al. 2010) revealed that the vulnerability of several provinces in sub-Saharan Africa led to poor adaptability among regions to effects of climate change owing to poor technical performance and technical incapacity resulting from overdependence on dry land farming. Such dependence leads to extreme food shortage and unemployment. These events made people look for refuge in fish farming which is vital for stimulating food security and generating household income. However, the significant contributions made by fish farming in Zimbabwe are being constrained by a myriad of factors, which include, *inter alia*, climate and hydrology, high risk and investment costs, increasing demand and market value for fish poor fisheries mismanagement, underfunding, poor fishery and aquaculture practices, and poor participation by communities and the environmental and social impact of aquaculture projects (FAO 2009). This article interrogated the effectiveness of fish farming in Mwenezi district to see if employment was created and food security improved among vulnerable households.

### Problem statement

The lack of economic opportunities and deteriorating climatic conditions in Mwenezi district, led communities to venture into fish farming as the only viable and economic alternative to mitigate poverty. Previously, subsistence farming, livestock management, bee keeping, gold panning, brick moulding, buying and selling have been dominant economic activities that were used by the people of Mwenezi although they fail to sustain because of poor economic conditions and drought induced by climate change (Chazovachii et al. 2013). The government of Zimbabwe acknowledges the rising poverty in the district and permitted NGOs to render fish farming projects in major dams such as Manyuchi and Musaverima in Mwenezi district. The prevalence of dry spells discourages domestic fish farming practices such as cage fish farming. Given the decline in agriculture because of the underperforming land reform programme among other rural livelihood activities, fish farming was implemented to help reduce poverty and dependency syndrome among rural communities. However, because of climate change which exacerbates droughts, limited funding and limited exposure to technology, fish farming does not entirely curb poverty but reduces the vulnerability of rural residents in Mwenezi district (Mufudza 2015). Some communities envision fish farming as an old trade for the elderly or less fortunate groups in communities, and hence they refuse to participate. Therefore, the article argues that effective implementation of fish farming as an innovative mechanism can generate employment and improve food security among vulnerable households in the drought risk district of Mwenezi. Nonetheless, population growth, biodiversity, deforestation, climate change and hydrology may inhibit the success of fish farming (Cook 2017). The article responds to the following research questions: What are the major factors affecting fish farming economic turnaround in Mwenezi district? What is the level of public participation in fish farming projects? Has fish farming mitigated high poverty levels and generated employment in Mwenezi district?

## Theoretical framework

### Resilience theory

Many authors provide different conflicting versions on the evolution of resilience theory. Van Brenda (2001:2) claims that resilience theory has been evolving over the past 70–80 years and subjecting itself to renaissance over the previous two or three decades. Greene et al. (2003) refer to resilience as a theory that can inform action. These authors regard the theory as a concept that changes people’s focus from the breakdown and disorder emanating from exposures to stressful environments, to individual characteristics and social processes associated with either normal or unexpected psycho-social development (Greene et al. 2003; Van Brenda 2001:2). The European Union Factsheets (2015) describes resilience as:

the ability of an individual, a household, a community, a country or a region to withstand, cope, adapt, and quickly recover from stresses and shocks such as violence, conflict, drought and other natural disasters without compromising long term development. (p. 2)

Crawford et al. (2005:355) add that resilience involves the search for knowledge about the processes which can contribute to positive adaptation and development in the context of adversity and disadvantage. Brand and Jax (2007) explain resilience as a concept embedded in sustainability that is applied in various disciplines to understand the socio-ecological systems. Often resilience theory is used to understand how multi-faceted systems change and how they withstand any disturbances that may occur (Janssen et al. 2006). From the aforementioned assertions, Walker et al. (2002) define resilience as the social-ecological system that has the potential to absorb disruptions and restructure itself while experiencing a change in order to maintain its function, identity, feedback and structure. Therefore, resilience in social-ecological systems involves several techniques that are used to adapt to change, stressors and sudden shocks (Adger et al. 2005).

In this article, the resilience theory has been adopted to explain ways in which fish farmers in Mwenezi district dealt with challenges arising from their own environments. The theory is fundamental as it draws on the remembrance of ways and strategies which had been used over time to try and cope with challenges affecting the livelihoods of rural people in drought risk regions (Lin 2011; Maleksaeidi & Karami 2013; Nelson & Stathers 2009; Neubert, Kömm & Krumsiek 2011). Resilience theory plays a major role in achieving sustainable fish farming as social and ecological capacities given the failure of other rural economic livelihoods such as livestock rearing and agriculture (Maleksaeidi & Karami 2013). Given the effects emanating from climate change, the theory strengthened fish farmers in Mwenezi district to resist any shocks and stresses such as Elnino-induced droughts which may disrupt their livelihoods; hence, fish farming can generate employment and improve food security among households.

### Global nature of fish farming

Gupta, Bartley and Acosta (2004) state that in Asian countries, aquaculture has been practised as part of integrating farming systems that are well incorporated into the local environment and available resources. In India, Ecuador, Indonesia, Bangladesh and other more established shrimp farming countries such as China and Thailand, fish farming has been widely practised and it contributes to about $6.3 billion in exports. A study by FAO (2016) supports the above assertion that Asia boosts the largest fisheries and aquaculture operations. The rich history of fish farming in China positions the country as the biggest world exporter, producing about 60 billion tons. Africa is also being regarded as the second continent in aquaculture yield per capita in the world (Cook 2017). The United States also acknowledges the benefits of the fish farming industry; hence, it is in the process of developing sustainable fish farming programmes. However, Gupta et al. (2004) condemn the growth in shrimp culture, a type of fish farming which has caused environmental and social problems such as destruction and conversion of farmlands into aquaculture ponds, pollution, salinity incursion and disease outbreaks. International literature asserts that fish farming contributes to Blue Economy or Blue Growth of the new maritime Green Economy. The UNEP (2011) reiterates further that fish farming is perceived as a Green Economy in a Blue World. FAO (2013) asserts that it is known as Blue Growth or Green Growth in Fisheries and Aquaculture. The growth in fish farming in the recent past can be regarded as a paradigm shift from other food security programmes towards a sustainable management of natural marine and freshwater resources. In developmental circles, of the green economy, the blue economy as a concept has been criticised because it focuses more on conservation and environmental protection, neglecting economic social development and economic growth.

### Fish farming in Africa

In sub-Saharan Africa, fish farming has quickly gained momentum as an innovative and economic mechanism for generating employment and increasing household income. According to FAO (2013), Tanzania presents the most favourable fish farming opportunity, supported by the abundance of land and water sources. About 14 100 freshwater fish ponds are available in Tanzania which are still to be tapped. Chenyambuga, Madalla and Mnembuka (2012) reiterate that aquaculture in Tanzania is still being operationalised at a subsistence level by small-scale farmers of low status although they are been constrained by lack of technology. FAO (2013) argues the lack of capacity in the government to exploit the viable fish farming which can be diversifying production and developing the export market for the largely rural Tanzanian economy. In Uganda, a study was conducted by Maurice, Knutsson and Gestsson (2010) to investigate the value chain of farmed African catfish. Similarly, Kariuki (2013) explores more on fish farming implementation in Kenya. The research explores the existing fish farming – mainly catfish farming – industry with its value chains. These studies responded to questions that arose in relation to fish farming culture, value chains, value distribution and how these links profit farmers and small fish farming commercial businesses. In Nigeria, Ike and Onuegbu (2007) devised aquaculture technology packages for Nigerian farmers. The intervention strategy was aimed to mitigate low technology levels in fish farming because of limited funding. Cook (2017) observes that fish farming has proven to be an innovative strategy for improving economic growth and well-being of communities. With a World Aquaculture Conference being held in the South African city of Cape Town, fish farming will dominate on how it can increase food supply and improve the economy of the country.

### Fish farming in Zimbabwe

At the launch of Zimbabwe Fish Producer’s Association (ZFPA) at Exhibition Park on 17 March, aquaculture or fish farming was integrated into agriculture as part of the livestock sub-sector. Fish farming was regarded as a livestock enterprise that has the potential to be developed into a full-fledged agricultural industry in Zimbabwe (Aquafeed Staff 2016). The EU-funded SmartFish programme is currently developing capacity building strategies to advance fish farming in the country. The fish farming industry in Zimbabwe is growing rapidly and has the potential to be developed into a major industry as Zimbabwe boasts 60% of the dammed water in the Southern Africa Development Committee (SADC), and possesses the largest freshwater fish farm in Africa. About 5% of the 400 000 hectares is being actively used for fish farming whereas commercial tilapia operations are vibrant in Lake Chivero, Kariba and Darwendale. However, the potential of fish farming in Zimbabwe is still largely untapped as noted by McCollum, the Chairman of ZFPA. The recent development in specialised fish feeds has seen tilapia growth rates rising for indigenous fish suited to high stocking in dams and ponds. The establishment of Aquaculture Quality Control Laboratory in Harare enables fish farmers to receive training in aquaculture value chain, marketing, trade and business development. Although fish farming is still growing at a slow pace in Zimbabwe, networking with other African countries (Kenya, Tanzania, Ghana, Namibia and South Africa) that are flourishing in fish farming is key to regenerating employment and boosting food security (Mapfumo in Acquafeed Staff 2016).

### Fish farming, food security and climate change in drought risk regions

As alluded in the introduction, the article examines fish farming as an innovative strategy for employment regeneration and food security in drought risk regions of Zimbabwe. Fish farming provides remarkable contributions to global food security and nutrition. Hall et al. (2011) contend that fish farming ensures food security by producing some low-value freshwater species which stimulate domestic production through integrated farming. The Conservation International and World Fish Centre (2010) conducted a global study which assessed fish farming in 18 different countries. Findings from the study revealed that fish farming is key to feeding growing urban populations. Results reflected further that fish farming and its fishery products provide valuable sources of protein and micronutrients for nutrition and good health. FAO (2012) claims that fish serves about 3.0 billion people with food in the world. Given these assertions, fish farming is innovative in boosting global food security and improving the household income of communities. Furthermore, the effects of global warming and climate change have serious consequences on fish farming communities in the local and global scene owing to overpopulated coastal regions (Ababouch & Fipi 2015). The risk of climate change is spreading fast to low lying areas, in the process causing loss of livelihoods, human displacement and migration. Also, floods and storms affect fish farming distribution in some regions, leading to unemployment and reduction in trade. World Bank (2013) warns that the effects of climate change can shift the status quo of people in tropical countries and can harm fish farming which is a livelihood for people living under vulnerable conditions. The article argues that the vulnerability of communities to climate change affect fishery resources and degrade ecosystems which is a hindrance to food security and employment creation in tropical and drought risk regions. Ababouch and Fipi (2015) therefore advise African governments to establish policies that maintain fish stocks, which is paramount for employment creation, food security and sustainable development. Africa’s capacity constraints should be harnessed to improve global cooperation in marine ecology and sustainable fisheries which are fundamental for improving global food security.

### Challenges of fish farming as an innovative strategy for promoting food security

The sustainable harvesting of fish stocks has ecologically determined upper limits for increasing the contribution of fish farming towards enhancing the standards of living for many rural dwellers (World Fish Centre 2011). Many developing countries are failing to fully realise the benefits of fish farming towards improving rural livelihoods because of overexploitation of resources, thereby reducing fish harvests (FAO 2010). The World Fish Centre (2005) argues that there is a need for maintaining equilibrium between exploitation and the benefits as a way of avoiding resource misuse. Precautionary measures and policies must be put in place to mitigate extinction of fish species and improving the harvest of fish farmers (Chenje 2011). Fish farming as an economically viable sector for employment generation is often affected by trends in the international, national and local environment. This can include inflation, economic downturn which discourages markets, and policies. Fostering favourable policies that promote fish farming should be government responsibility in order to generate employment and improve the economy of the country (Thorpe 2012). In Zimbabwe, the limited success of fish farming is often attributed to limited funding, technology and poor implementation of fish farming practises. Other factors such as pollution, deforestation, soil erosion, floods, land shortages, environmental degradation and lack of fresh water sources affect the breeding of fish. FAO (2009) advocates for policy formulation to safeguard small-scale fisheries that are fundamental for improving rural livelihoods. Stakeholders are challenged to provide support to fish farmers in vulnerable provinces where fresh water is abundant in order to generate employment and promote food security in such regions.

## Materials and methods

### Design

A research design is described as a plan used by a researcher to obtain participants (subjects) and collect information from them (Welman, Kruger & Mitchel 2005). The article used a qualitative research design consisting of 12 semi-structured interviews administered to fish farmers and officials from the NGO practising aquaculture (fish farming) in Mwenezi district in Zimbabwe. Creswell (2009) asserts that a qualitative research design explores and understands the meaning of individuals or groups ascribed to a social or human problem. The approach emanated from an interpretive paradigm that seeks to understand the hidden meaning concerning people’s behaviour and actions. Therefore, the qualitative research design was used to understand the views of fish farmers and whether employment was generated to improve their livelihoods.

### Setting

**FIGURE 1:**
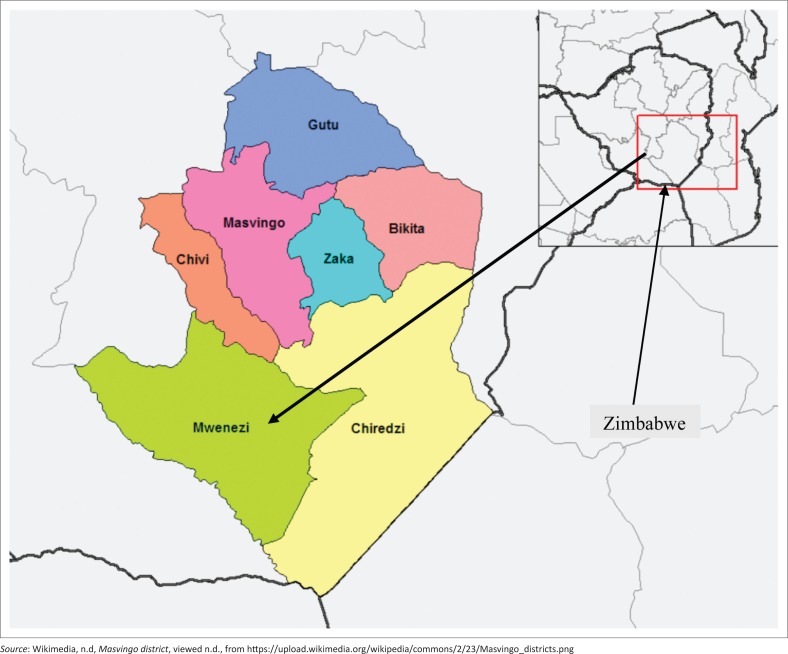
Map for Mwenezi district, Zimbabwe.

The article assesses the viability of fish farming as an innovative and economic mechanism for promoting food security in Mwenezi district in Zimbabwe. Zimbabwe is divided into five agro-ecological regions depending on temperature, rainfall and altitude. Region I is found in the Highveld, with high rainfall of above 1000 mm per annum, while Region II receives 750 mm – 1000 mm per annum. Regions III, IV and V, receive 650 mm – 800 mm, 450 mm – 650 mm and less than 450 mm, respectively (Muchara 2010). Mwenezi district (Figure 1) is located in agro-ecological region IV, with an average rainfall of 540 mm per annum and highly abnormal temperatures above 25 degrees Celsius in the hot summer period. The natural region IV is a semi-extensive farming region covering about 38% of Zimbabwe. Rainfall is low and periodic seasonal droughts and severe dry spells during the rainy season are common. Mwenezi district therefore lies in a drought risk region IV and V which is mainly based on livestock production as crop production is risky except in certain very favourable localities where limited drought resistant crops are grown (Mafu 2011; Muchara 2010). Because of the nature of the district, fish farming evolves to utilise the largely unused water bodies as a way of generating employment and enhancing food security for the vulnerable households. The fact that the region receives low rainfall makes droughts rampant almost every year; hence, subsistence agriculture is done on a small scale because of poor climatic conditions. Based on the failure of other economic activities as alluded to in the literature, fish farming becomes an alternative technique to create jobs and household income for sustainable livelihood of the rural residents.

### Sampling technique

De Vos et al. (2011) state that purposive sampling technique falls in the category of the non-probability sampling method. It is based entirely on the judgment of the researcher. In this article, therefore, purposive sampling technique was used to select 12 participants from project members of whom 3 were programme officers from the three NGOs who indulge in fish farming in the case study. This sample represents the broader participants in the district who engaged in fish farming. The researchers conducted semi-structured interviews with programme managers and fish farmers (participants) which were triangulated with secondary data acquired from a desktop review. Documents related to fish farming from the European Union (EU), World Food Programme (FAO) and Aquaculture Zimbabwe were used to establish the existence of fish farming projects and determine the weakness and gaps for further consideration.

### Data analysis

Data acquired from interviews were transcribed verbatim and presented in themes following the flow of paper objectives. Content analysis was used to present secondary data. Peer reviewed articles were used to assess the reliability of research instruments. Content analysis as Neuman (2006) observes refers to a method for gathering and analysing the content of text while content refers to words, meanings, symbols, pictures, ideas, themes or any message that can be communicated and text is anything written, visual or spoken that serves as a medium for communication.

### Data trustworthiness of qualitative study

Guba and Lincoln (1982) ascertain that credibility, dependability, conformability and transferability are used to enhance the trustworthiness of qualitative research findings. For this article, credibility was achieved by using multiple sources that inform fish farming in the global and local scale. Dependability of results concerning fish farming was realised through employing peer examination. Conformability was tested using triangulation of observer and triangulation of methods (Anney 2014). Transferability was tested by applying a detailed description of the research context. This involves thoroughly describing the research methods and assumptions underpinning the study.

## Results and discussion

Fish farming as an innovative strategy for promoting food security in Zimbabwe is being constrained by various factors which may compromise food security in the drought risk district of Mwenezi. The researchers noted the following challenges that hinder fish farming.

### Improving capacity building and strategic management among non-governmental organisations

The participants revealed that NGOs under study faced various challenges in advancing fish farming in Mwenezi district. Findings revealed that NGOs under study had governance and management structures in place; however, the structures were abused by those at the helm of power. As a result, the mismanagement and resource misallocation was experienced as paving the way to the collapse of various fish farming projects. There were widespread corrupt tendencies in the management whereby funds meant for fish farming projects were sometimes diverted or were never used for empowering communities as they were intended to. The financial problems faced by the NGOs in fish farming projects in Mwenezi district posed serious threats to food security and employment creation in the drought prone district. Poor accountability mechanisms in the NGOs impacted negatively on employment creation and food security as other fish farming projects were liquidated because of a lack of transparency and accountability. These findings correspond with a study conducted by Lekorwe and Mpabanga (2007) which advocated for accountability and transparency in NGO operations as these factors are essential in enhancing good governance. Drawing analysis from these findings, therefore, the success of fish farming as an economic and innovative mechanism for improving rural livelihoods is hinged on proper strategic management which adequately utilises resources in an efficient and effective manner.

### The need to raise awareness on fish farming

The article establishes further that two of the NGOs lacked capacity building programmes which involve staff training and development through workshops and seminars to realise efficiency in aquaculture projects. The shortage of consistent capacity building programmes results in underproduction in fish farming projects. This was constrained further by insufficient funds to outsource organisations for training the NGO staff. The article observes that only one out of the three NGOs implemented capacity development programmes that were aimed at increasing staff performance in fish farming projects. This NGO established a vocational school to train the local people in technical fields ranging from carpentry to farming, building, weaving and most importantly fish farming. These programmes were meant to empower local people to be self-reliant and resilient to poverty in the drought risk area. Based on the findings capacity building remains a fundamental aspect for enhancing performance among NGOs indulging in fish farming.

### Rehabilitating small dams for fish farming

In an effort to create an enabling platform for fish farming in Mwenezi district, the three NGOs embarked on the progressive rehabilitation of small dams and conservation farming. The results of the article revealed the NGO sponsored projects such as small dam rehabilitation in Mwenezi East, Ward 2 and 3 in areas such as, Rata, Musaverima and Shazhaume just to mention a few. Small dam rehabilitation was essential in supplementing water supplies and promoting fish farming as Mwenezi district is a dry region because of periodic drought spells. One of the NGOs financed smallholder irrigation schemes in Ward 3 under Chief Mawarire. The NGO provided drip kits which were economically efficient for drip irrigation to communal farmers in the ward. The NGO further implemented conservation farming which is an adoption of several husbandry practises to improve farming. These strategies enable farmers to plant a large area as they are not moving or turning over the soil before they plant. Soon after harvesting farmers began preparations to plant early once rain falls. Labour is spread across the year; hence, farmers are flexible. In drought risk regions such as Mwenezi, conservation farming was vital for reducing crop loss and hence improving food security. This strategy remains one of the most efficient farming methods ever introduced by NGOs in the case study area. Although this article focuses on fish farming, the significance of subsistence agriculture is fundamental; hence, the two drought coping mechanisms complement each other.

### Fish farming and improved food security and nutrition

The implementation of fish farming in Mwenezi district by NGOs assisted in regenerating employment and improving food security and nutrition among community members. From the interviews conducted with one field officer from the NGOs indulging in fisheries and conservation of acqualife, it has been discovered that communities greatly enjoyed several rewards coming from aquaculture projects. The local people were sponsored with boats, nets and technical expertise on how to fish in major dams such as Musaverima and Manyuchi Dam, the biggest dam in Mwenezi district. Produce (fish) from this project is sold to local markets and some even exported beyond international boundaries. The article observes that the fish farming projects involve the farming of aquatic organisms, including fish, molluscs, crustaceans and aquatic plants. Fish farming as a poverty alleviation technique was very successful as food security and household income greatly improved. Kawarazuka (2010) confirmed in his study that fish farming contributed to food security and dietary improvement among households engaged in fish farming projects. The purchasing power for many households was improved as fish farming was profitable.

The field officer interviewed remarked that:

The day we launched the fisheries project many villagers became eager to join the fishing clubs within our project. The fisheries project was flooded since many people saw the benefits that were going to come out this project. Food security greatly improved as the produce (fish) was sold to the local markets and some exported at highly competitive prices. The project members were able to sustain their families and send their children to school. (NGO, Male Participant)

It can be deduced from this project that employment was created which improved the economic lives of the people in Mwenezi. However, a study by Brummett (2008) shows that there is a need to increase investment in fish farming to meet customer demand. Small-scale fish farming in Mwenezi district should therefore be increased to meet global markets which require adequate research and investment in fish farming projects.

### The need to obtain sustainable funding

The implementation of fish farming and other food security programmes in Mwenezi district was incapacitated by limited funding from the donor community. Findings reflected that donor agencies failed to adequately fund local NGOs which results in the selective implementation of fish farming projects as some communities were left marginalised. The challenges emanated from the high level bureaucracy in the donor community which requires strong accountability to boards of directors and government ministries that release funding.

One participant states:

Fish farming is a good poverty alleviation programme which is helping us in our villages. But ever since we joined the programme, we do not have adequate funding as the NGOs are running many fish farming projects resulting in us being underfunded. We appeal to the government and well-wishers to assist with funding to buy more materials and vehicles to transport our produce to nearby markets. (NGO, Female Participant)

Based on the financial challenges to fund fish farming, there is a need for NGOs to network and foster relations with other business people and financial institutions which can fund development programmes.

### The need to increase monitoring and evaluation of fish farming projects

The three NGOs under study admit to the shortage of financial resources which affect them in conducting Monitoring and Evaluation (M&E) on fish farming projects. The article affirms that M&E was regarded as an expensive exercise that required excessive funding to undertake; hence, the three organisations chose to ignore it on grounds of poor revenue base. The findings revealed that the shortage of funds hindered the NGOs from hiring external evaluators that could have bankrupted their already bleeding accounts. The findings highlighted on the shortage of technology (i.e. computers) and technical expertise among NGOs’ employees which could have posed serious challenges in implementing M&E strategies. The participants from all NGOs openly state that many NGO staff members were technologically inefficient which shows weaknesses in managerial leadership or. Based on these scenarios the NGO top management was supposed to initiate training programmes which help to equip their staff with the necessary skills to cope with the trends in the economic and technological environment. A study by Belton and Little (2011) endorsed the findings when it revealed that external interventions on fish farming projects are important for sustainability and resilience of households in vulnerable communities.

### The need to earn legitimacy in fish farming projects

The success of NGOs in Zimbabwe rests on their ability to earn legitimacy with the people and government. The article revealed that legitimacy and political interference from government have been two major setbacks affecting fisheries and food security projects in Mwenezi district. NGOs indulging in fish farming projects are required to seek approval from traditional leadership (Chiefs, Village Herds) and local municipality which often derails this poverty reduction strategy.

One NGO Programme Officer states that:

Before we started fish farming projects we sought permission from the Council and Local Leaders to hold community meetings with the people. The reason was to earn legitimacy so that local people can understand and believe in our mission. We had to wait for some days whilst our application was being processed. The long period of waiting disappoint some communities who were waiting for the fish farming projects to start. (NGO, Male Participant)

These findings augur well with a ban imposed on NGOs in Masvingo province in 2013 by the Provincial Governor who accused them of being Western Imperialists who are driving an anti-government agenda under the mask of development aid (Chitagu 2013). Although obtaining legitimacy proved to be a stumbling block in fish farming projects, other NGOs in the case study area engaged in poverty alleviation and resumed operations as they were regarded as grassroots and Christian centred organisations.

### Fish farming and improved household income

Fish farming was implemented in the study area to regenerate employment and improve the income for rural people. The participants of the study revealed that fish farming has emerged as an alternative and fast money making spinner to transform their livelihoods.

Participants from one fish farming project explain that:

The inception of fish farming in our district enabled us to improve our lives as we supply fish to every store, schools and food organisations in our area. The demand for fish is too high that we are failing to satisfy customer needs. The fish farming project has helped us send our children to school and improved our lives greatly as food in our homes is always in abundance. We appreciate the efforts done by the NGOs and we ask them to continue providing us with technology for fish farming so that we become self-dependant in providing food for our families. (Male, Community Member)

The above assertions clearly show that participants embraced fish farming which was fundamental for employment creation, improving food security and household income to meet basic needs. Findings reflected further that NGOs in Mwenezi undertook an active role in mitigating high poverty levels in the district although there is a need to instil a sense of complete ownership among community members so that full participation is achieved for sustainable development.

### The need to improve market opportunities

The success of any viable entrepreneurial venture rests on the availability of markets to export the produce. The article establishes that the lack of market opportunities and low cash injections hindered fish farming in Mwenezi district. The growing competition of fish farming in Zimbabwe saw markets becoming flooded owing to the decline in the economy, and so the businesses are fast losing their profits. The shortage of ready markets led to fish farmers selling fish to local neighbourhoods and businesses at a relatively low price because of unavailability of the external markets. Failure to obtain ready markets constrained fish farmers to expand into a more commercialised business; hence, they remain small at subsistence level. The majority of participants pointed to the lack of credit facilities to finance labour and technologies as NGOs often took time to provide the needed equipment to use in fish farming projects.

One of the participants laments:

After harvesting fish we face challenges in terms of transport and markets to sell our produce. The local businesses and growth points what are our main target are always full since the competition is very high in our district. We end up selling our fish to individuals such as teachers, business owners at low prices which is discouraging since fish farming is hard work and we sometimes get little. We do not have enough knowledge about where to sell our fish as other towns are very far and we do not have enough money to hire trucks to transport the fish to other big towns. (Female Community Member)

The reason is that the participants who are of rural origin lack collateral security to solicit loans from local banks such as Agricultural Bank (AGRIBANK). The lack of borrowing power often exposes rural people to donor dependency syndrome. This limited access to markets was acknowledged in a study by Wetengere (2011) where fish farmers in Tanzania are finding it difficult to find markets for exporting their fish. The article further questions the dependency syndrome among local NGOs as detrimental to societal development. Based on these findings it can be argued that there is a need for NGOs to establish a sustainable funding model to finance aquaculture projects which are a fundamental step in promoting food security in the district.

### Improvements in technology in fish farming projects

The trends in the technological environment globally require organisations to adopt and cope with a wave of transformation which can improve the viability and profit margins of a business. The participants cited the limited technology application in the fish farming projects. This reduces the opportunity of fish farming to flourish into a more commercialised sector. On the issues of technology usage in fish farming, the participants lacked proper storage facilities, hatchery and improved fish feeds among others. The article points out further that fish farming in Mwenezi district has shown the need for extension services; however, there is inadequacy or non-viability of the extension services to disseminate information on the proper use of medicines, suitable fish farming technology and good management practises for fish farming. Research by Adinya, Offem and Ikpi (2011) supports the results when it endorses that fish farming projects fail because of limited skills to use modern technology. The results of the study reflected that there was a huge skills gap which inhibited fish farming to expand which calls for education provision for fish farmers to enhance food security and employment creation. Based on these findings, it remains a challenge to NGOs implementing fish farming in Mwenezi district to implement latest technologies that enhance production and increase income and food security among vulnerable households.

## Ethical considerations

According to Myer (2009), ethics involve the application of moral principles when planning, conducting and reporting the results of research studies. Stangor (2011) endorses that ethical behaviour in science includes honesty not only in conducting research, but also in reporting it and giving proper credit for ideas. In this article as McNabb (2002) observes, four practical ethical principles are most relevant to research in social sciences and these principles are truthfulness, thoroughness, objectivity and relevance. The researchers observe the above four principles in presenting data concerning fish farming in the case study area. Informed consent for participants was obtained; hence, no one was forced to participate in the study. Ethical clearance was sought from the University of Fort Hare and permission was granted as well by the Mwenezi district Administrator. The article avoided plagiarism and fabrication of data; hence, the findings were reported in an honest and academic manner.

## Discussion

The findings of the article show that NGOs in Mwenezi district have been playing a vital role in fish farming projects that were regenerating employment and increasing household income and food security. Nonetheless, limited technological application hinders the transformation of fish farming into a more commercialised income generating venture. NGOs are often regarded as the third sector in the economy of the country and have been advancing several innovations and new models that have been adopted by various stakeholders in developmental circles. Because of their generally flexible organisational structures and characteristics such as organisational autonomy and participatory structures, NGOs have added value to fish farming projects in Mwenezi district as they have abundant time to engage relevant stakeholders such as community members to discuss and teach them the nature and way to self-sustain in the drought risk district. Therefore, fish farming as an innovative mechanism for improving food security in the drought stricken district led to socio-economic improvements in most rural areas under study. Nevertheless, NGOs need to improve their managerial capacities to achieve effective food security and employment creation among vulnerable households in Mwenezi district.

## Conclusion

The article observes that the implementation of fish farming projects by three NGOs in Mwenezi district has been widely embraced as it helped reduce the dependency syndrome among rural communities by generating employment for sustainable food security. Various factors such as poor infrastructure, limited market opportunities, shortage of finance, lack of research training extension, M&E and legitimacy affect the NGOs in the district. However, the study has shown a positive relationship between employment regeneration and improved food security through fish farming. As a drought prone area, fish farmers in Mwenezi district have since improved their household income; hence, fish farming can be regarded as an innovative and economic mechanism for transforming rural livelihoods.

## Limitations of the study

The study faced various challenges ranging from the remoteness of the study area, time constraints and limited financial resources as it involves traveling to fish farming projects within the district. The other main constraint was the reluctance by other participants to respond to interview questions. To mitigate these challenges, some friends and family assisted with funding to be able to finish this study.

### Recommendations

Based on the conclusions drawn from this article, NGOs need to improve on their research culture to widen the scope of fish farming, for instance implementing cage fish farming which is easy to manage. Public-Private Partnerships (PPPs) are vital for NGOs to subsidise fish farming and offer incentives to feed production and processing industries as fish farming as an economic sub-sector improves food security, nutrition and creates employment. The government needs to support fish farmers with access to credit facilities to finance aquaculture projects. NGOs need to provide skills training on fish farming to rural communities to bridge the technology skills gap. The contribution of fish farming to the economy of Zimbabwe needs to be embraced by including it the national budget as a viable poverty alleviation technique.
